# Evaluation of Long-Term Clinical Outcomes and Patient Satisfaction Rate Following Low Intensity Shock Wave Therapy in Men With Erectile Dysfunction: A Minimum 5-Year Follow-Up on a Prospective Open-Label Single-Arm Clinical Study

**DOI:** 10.1016/j.esxm.2021.100384

**Published:** 2021-06-11

**Authors:** Eric Chung, Ross Cartmill

**Affiliations:** 1 The University of Queensland, Department of Urology, Princess Alexandra Hospital, Brisbane, QLD, Australia; 2 AndroUrology Centre, Brisbane, QLD, Australia; 3 AndroUrology Centre, Sydney, NSW, Australia

**Keywords:** Low Intensity Extracorporeal Shock Wave Therapy, Erectile Dysfunction, Clinical Outcomes, Patient Satisfaction, Long-Term Data, Erectile Function

## Abstract

**Introduction:**

Low intensity extracorporeal shock wave therapy (LIESWT) improves erectile function (EF) in men with vascular erectile dysfunction (ED) but longer-term outcomes remain unknown.

**Aim:**

To evaluate the clinical outcomes of LIESWT at a minimum 5-year follow-up.

**Methods:**

This is an open-label single-arm prospective study involved men with vascular ED who received LIESWT.

**Main Outcome Measure:**

Changes in patient demographics, IIEF-5 and Erectile Dysfunction Inventory of Treatment Satisfaction (EDITS) scores, as well as overall satisfaction score (on a 5-point scale) were reviewed at 12, 24, 48, and 60 months after completion of LIESWT. A chi-square contingency analysis was used to examine the relationship between erectile function score and treatment satisfaction, with statistical significance set at 5%.

**Results:**

The mean follow-up period was 69.9 (63–82; median 76) months. The mean IIEF-5 scores for pretreatment and after treatment at 12, 24, 48, and 60 months were 14.8, 17.6, 16.8, 16.5, and 16.5 while the percentages of patients who reported an improvement in IIEF-5 score by 5 points were 60%, 45%, 40%, and 40%; and EDITS scores >50% were recorded in 70%, 55%, 50%, and 48% of patients at 12, 24, 48, and 60 months post-LIESWT. Ten patients required medical therapy and 2 patients opted for penile prosthesis implantation. The overall satisfaction rate appeared sustained subsequent follow-up (score 4 out of 5; 68% vs 50% vs 40% vs 40% at 12, 24, 48, and 60 months). There were minor time-limited, but no significant adverse event reported.

**Conclusion:**

This long-term study showed the observed clinical improvement in EF continues to deteriorate but appears to plateau at 40% clinical efficacy at 48–60 months after completion of LIESWT. The absence of penile pain and deformity at 5-year follow-up supports the long-term safety data of LIESWT in men with ED.

**Chung E, Cartmill R. Evaluation of Long-Term Clinical Outcomes and Patient Satisfaction Rate Following Low Intensity Shock Wave Therapy in Men With Erectile Dysfunction: A Minimum 5-Year Follow-Up on a Prospective Open-Label Single-Arm Clinical Study. Sex Med 2021;9:100384.**

## INTRODUCTION

The landmark article by Vardi et al^1^ reignited the interest in the role of low intensity extracorporeal shockwave therapy (LIESWT) to treat men with erectile dysfunction (ED). Given the positive effects shown in various animal studies to support the role of LIESWT in penile neovascularization,^2^ this therapy can potentially reverse the underlying ED and transform the current ED treatment landscape. Published systematic review and meta-analyses showed encouraging clinical outcomes in men with ED.^3^, ^4^, ^5^, ^6^, ^7^ Zou et al^4^ reported LIESWT was 8.31 [95% confidence interval (CI): 3.88–17.78] times more effective than sham therapy in improving erection based on erection hardness score (EHS), and 2.50 (95% CI: 0.74–8.45) times in terms of erectile function scores. Based on extracted data from 7 clinical trials, Clavijo^5^ found a statistically significant improvement in pooled change in the International Index of Erectile Function (IIEF) score compared to sham group (6.40 points; 95% CI: 1.78–11.02; *I*^2^ = 98.7%; *P* < .001 vs 1.65 points; CI: 0.92–2.39; *I*^2^ = 64.6%; *P* < .001). Similarly, Lu^6^ analyzed 14 studies and showed LIESWT significantly improve IIEF (mean difference: 2.00; 95% CI: 0.04–0.29; *P* = .01) and EHS (risk difference: 0.165; 95% CI: 0.04–0.29; *P* = .01).

In the past year, several sexual medicine societies have adopted LIESWT as a cautious treatment with reasonable clinical efficacy and safety to treat men with ED but should be undertaken in the setting of clinical research.^8^^,^^9^ The pooled data from meta-analyses including RCTs showed an overall positive effect in terms of IIEF-EF score improvement, although the estimates are small (ranging from about 2–4 points of the IIEF-EF) and the heterogeneity high.^8^ Most published studies did not extend beyond 2 years follow-up^9^ and the question arises whether the observed early improvement in EF can be sustained in the longer-term.^2^^,^^3^, ^4^, ^5^, ^6^, ^7^ Furthermore, clinical safety data on the impact of shock waves on the penile tissue over a longer period are yet to be fully elucidated. To our knowledge, this is the first long-term clinical study that prospectively evaluates clinical outcomes and patient satisfaction rate following LIESWT in men with ED at a minimum 5-year follow-up. The hypothesis of this study is to determine if observed clinical efficacy and safety of LIESWT remain similar in the long-term?

## METHODS AND MATERIALS

All patients completed the LIESWT treatment in 2014 and the original study methodology and clinical outcomes were published in 2015.^10^ In brief, patients received 3,000 shocks (1,000 shockwaves to the distal penis, 1,000 shockwaves to the base of penis, and 500 shockwaves to each crura on the perineum) using Duolith SD1 ultra (Storz Medical AG, Tägerwilen, Switzerland) at an energy density of 0.25 mJ/mm^2^ and emission frequency of 6 Hz, twice weekly for 6 weeks. This study has received internal departmental ethics approval and clinical data were collected in a prospective manner. An improvement ≥5 points from the baseline IIEF-5 score is considered significant based on change in the severity of ED category.^11^

All patients in the original study were prospectively reviewed at 12, 24, 48, and 60 months after the completion of LIESWT. All patients attended regular appointment as part of their routine annual Men's Health check-up and phone calls were made to contact patients who did not attend the clinic review in person. Patient demographics, changes in IIEF-5 and Erectile Dysfunction Inventory of Treatment Satisfaction (EDITS) scores, and overall satisfaction rate (on a 5-point scale) were collected and updated. Treatment-related adverse events such as penile pain, bruising, hematuria, and subsequent penile deformity were recorded too.

Statistical analysis was performed with SAS 9.1.3 (SAS Institute, Cary, NC, USA) computer software with values of the study parameters compared using Student *t*-test or Wilcoxon signed-rank test as appropriate. A chi-square contingency analysis was used to examine the relationship between erectile function score and treatment satisfaction, with statistical significance set at 5%.

## RESULTS

### Patient Demographics

A total of 30 patients were recruited in the original study and no patient was lost in this follow-up study. The mean age at the time of LIESWT was 55.8 (42–68; median 48) years and the majority of men (80%) had ED for more than 18 (mean 21.8; 6–60) months. The mean follow-up period was 69.9 (63–82; median 76) months.

Cardiovascular risk factors were present in 26 patients with 10 patients reported previous ischemic heart disease and 10 patients suffered from diabetes mellitus. The etiologies for ED were vasculogenic (27) and radical prostatectomy (3). The mean IIEF-5 score was 14.8 (12–18) and the majority of patients have a stratified moderate ED classification (60%). At the minimum 5-year follow-up, 4 additional men developed cardiovascular risk factors (4 men were diagnosed with hypertension and 2 men had hyperlipidemia).

### Efficacy, Safety, and Patient Satisfaction Rate

**Figure 1:**
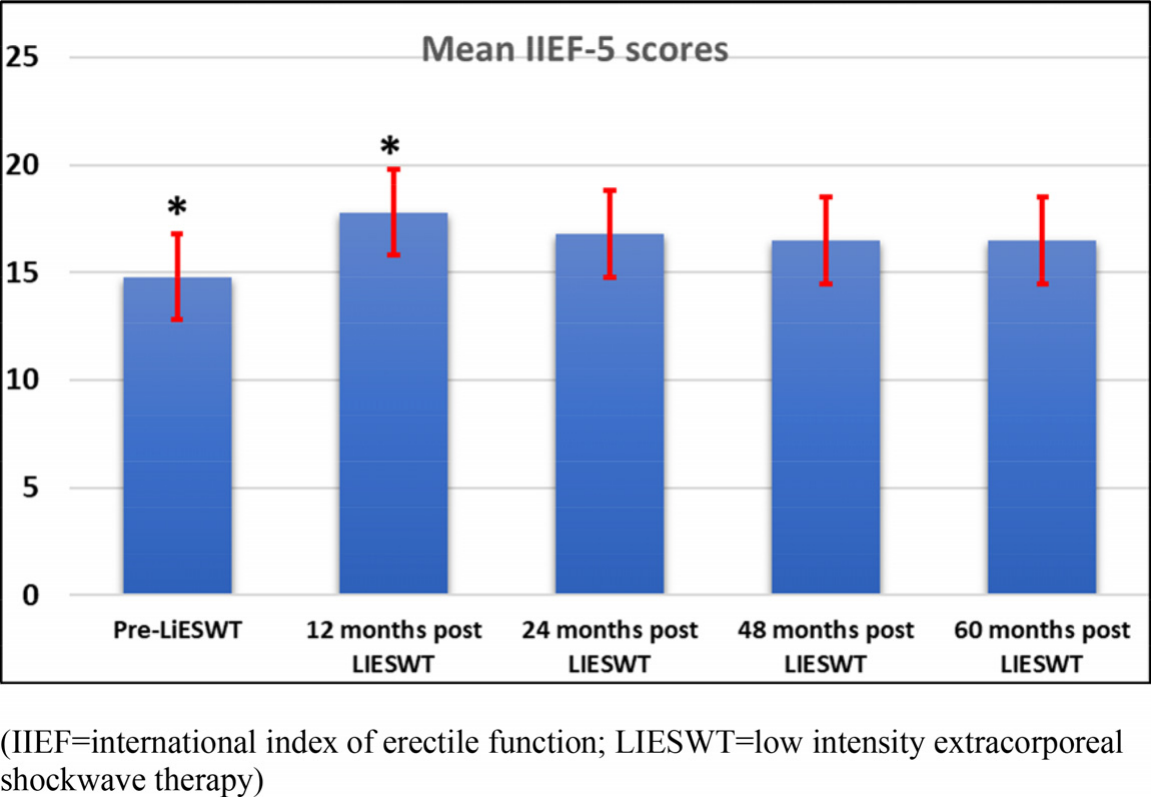
Comparison of the mean IIEF-5 scores following LiESWT across various periods following LIESWT completion. Asterisk indicates *P* < .05 and represents significance of difference between the 2 groups. IIEF = International Index of Erectile Function; LIESWT = low intensity extracorporeal shockwave therapy.

The initial study showed a total of 18 (60%) patients reported at least 5 points improvement in IIEF-5 score and 21 (70%) patients recorded an improvement in EDITS Index score >50% at 4 months follow-up study. In comparison to initially published outcomes, the reported improvement in EF was not sustained. The mean IIEF-5 scores for pretreatment and after treatment at 12, 24, 48, and 60 months were 14.8, 17.6, 16.8, 16.5, and 16.5 (see Figure 1). The percentages of patients who reported an improvement in IIEF-5 score by 5 points or more were 60%, 45%, 40%, and 40% of patients at 12, 24, 48, and 60 months following completion of LIESWT. The EDITS scores >50% were recorded in 70%, 55%, 50%, and 48% of patients. There was no significant difference detected in EF decline between 48 and 60 months (*P* = .44). Ten patients required medical therapy and 2 patients opted for penile prosthesis implant. Five patients have elected to undergo a second cycle of LIESWT and further improvement was observed in 3 men with a return of EF to the previous state (ie, IIEF-5 score >21). Similar to the initial clinical findings, a sustained improvement in EF was seen in men with vasculogenic ED and not in those who underwent radical prostatectomy (*P* < .05).

At the initial study period, two-third of patients scored 4 out of 5 in overall satisfaction, and this overall satisfaction rate appeared to decrease in the first 2 years but remained stable on the subsequent follow-up after 2-year post-LIESWT (68% vs 50% vs 40% vs 40% at 12, 24, 48, and 60 months). There was a positive correlation between men who reported improvement in EF and treatment satisfaction level with LiESWT (*P* < .05).

Similar to the initial publication, there was no reported treatment-related adverse event across the follow-up interval period. No patient developed penile pain or deformity at a minimum 5-year follow-up.

## DISCUSSION

The ideal treatment for ED should restore EF to spontaneous erection permanently without any adverse effects. The LIESWT is a noninvasive and safe treatment that can restore natural and spontaneous EF by improving penile hemodynamics and underlying pathological changes through its angiogenic properties.^2^^,^^12^ Over the last few years, the use of LIESWT for ED has gained considerable popularity and is rapidly adopted and endorsed as an effective treatment in a carefully selected group of men with ED.^8^^,^^9^ Various shockwaves machines with different energy composition have been utilized, and numerous studies were undertaken with good clinical outcomes.^2^

The longer-term physiological effect of LIESWT on EF recovery is largely unknown. While basic science research confirms that LIESWT stimulates the release of various angiogenic and neurotropic factors resulting in regeneration of cavernosal smooth muscle and endothelium,^2^ these effects may not be permanent. Given that natural progression and regression of ED are common over a longer period of observation,^13^ coupled with the fact that some men will likely accumulate more cardiovascular risk factors resulting in endothelial dysfunction, it is foreseeable that the positive effects of LIESWT may not be long-lasting. The majority of published studies on EF recovery following LIESWT have been limited to 1 year of follow-up^3^, ^4^, ^5^, ^6^, ^7^ with reported positive clinical improvement in EF scores and statistically significant improvement in the pooled change in IIEF score compared to the sham group.^5^^,^^6^ While our initial publication mirrors contemporary literature, the early clinical improvements in EF were not sustained in the longer-term. Compared to the initial study publication with 60% of men reported an improvement in IIEF-5 score by 5 points, these percentages decreased to 45% at 24 months before reaching a plateau of 40% at 48 and 60 months following completion of LIESWT. Similarly, the deterioration in EDITS scores >50% were observed with a significant deterioration between 12- and 24-month follow up (70% vs 55%). This is reflected in the decline in patient overall satisfaction rate, although there remains a positive correlation between men who reported improvement in EF and treatment satisfaction level with LiESWT (*P* < .05).

Patient selection appears paramount to treatment success and patients with mild–moderate ED, younger age group, those with minimal cardiovascular comorbidities, and the absence of diabetes or cavernous nerve injury are likely going to report higher EF recovery and spontaneous erection.^8^^,^^9^ In our study, men with vasculogenic ED responded better to LiESWT than men who developed ED following radical prostatectomy (*P* < .05). It is possible that the presence of corporal hypoxia resulted in higher expression of profibrotic factors thereby limiting the effects of LiESWT in terms of cavernosal neovascularization and neuroregeneration. A more recent study by Baccaglini et al^14^ reported that the improvement in IIEF-5 score was not clinically significant to support the role of LIESWT as penile rehabilitation, and further exploratory analysis found no significant difference between groups with an IIEF-5 score ≥17 (17.1% vs 22.2%; *P* = .57).

Presently, the actual physiological changes in penile tissues in the long-term remain largely unknown. Our unit published one of the earliest studies on the use of Duolith SD1 machine for ED, and the electromagnetic shock wave can be delivered at the maximal energy density of 1.25 mJ/mm^2^ at 65 mm penetration depth. We did not find any adverse effect of LIESWT at the initial study period nor at 5-year follow-up, regarding penile pain and/or development of penile deformity. In contrast to the theory of microtrauma in the pathogenesis of Peyronie's disease,^15^^,^^16^ LIESWT may not contribute to penile plaque formation due to its regenerative properties with the release of vascular endothelial growth factor and endothelial nitric oxide synthase which are responsible in tissue angiogenesis.^17^^,^^18^ Furthermore, LIESWT has been shown to recruit endogenous mesenchymal stem cells, which has beneficial effects for the repair of damaged tissue.^19^ In an animal experiment based on diabetic ED model, LIESWT appears to restore underlying fibromuscular pathological changes and endothelial dysfunction within the corpus cavernosum.^20^ However, we agree that further research should be conducted to examine various pathophysiological alterations related to LIESWT on penile tissue including actual histological changes in the longer-term.

We acknowledged several limitations to our study include small number of participants, the lack of a placebo treatment arm and absence of objective penile hemodynamic measurements such as penile color Duplex ultrasonography. However, there is a strong correlation between the subjective report of EF recovery and objective penile hemodynamic improvements as demonstrated by previous studies.^2^ Any potential “placebo effect” (given the lack of a comparative placebo arm in the original study^10^) would have dissipated beyond 12 months review so the reported improvement in erectile function is likely a true effect of LIESWT. The use of validated questionnaires and complete data collection at a minimum 5-year period in a prospective manner further validated our initial clinical findings.^10^ While our treatment template is based on manufacturer's guidelines and likely derived from previous orthopedic research, many LIESWT studies have adopted our treatment protocol and showed similar successful outcomes.^21^, ^22^, ^23^, ^24^ Based on our long-term study findings, we conclude the positive improvements in EF following LIESWT is not sustained and there was with a gradual decline in EF scores before reaching a plateau after 48 months. We recognized that further dose finding LIESWT study comparing various treatment protocols and different shock wave machines should be conducted to improve treatment delivery and efficacy. Hence, mainstream sexual medicine organizations advocate a cautious approach to the adoption of LIESWT and that this regenerative technology should be offered to patients following adequate informed consent and usually undertaken in the context of clinical trial.^8^^,^^9^^,^^25^ Randomized clinical studies on the use of adjunctive measures such as cellular-based technology to augment the clinical effects of LIESWT will be useful and can add to the current armamentarium to treat ED.

## CONCLUSION

The potential role of LIESWT as regenerative therapy in penile rehabilitation to cure ED, unlike most conventional ED treatments, is exciting and novel. However, patients should be counseled that the immediate improvement in EF may not be sustained since this long-term study shows an observed initial decline in the EF recovery after the completion of LIESWT, before reaching a plateau at 48–60 months. This coincides with lower patient satisfaction rates over years. Nonetheless, the absence of penile pain and deformity at 5-year follow-up supports the long-term safety data of LIESWT in men with ED.

## Statement of Authorship

Eric Chung: Conceptualization, Methodology, Data Analysis, Draft, Review & Editing, Final Approval; Ross Cartmill: Methodology, Draft, Review & Editing, Final Approval.
